# An Overview of the First Use of the Terms *Cognition *and *Behavior*

**DOI:** 10.3390/bs3010143

**Published:** 2013-02-07

**Authors:** Daniel W. Chaney

**Affiliations:** Humanities and Social Sciences Division, University Libraries, Oklahoma State University, 306 Edmon Low Library, Stillwater, OK 74078, USA; E-Mail: dan.chaney@okstate.edu; Tel.: +1-405-744-9772; Fax: +1-405-744-7579

**Keywords:** cognition, behavior, history, origin, appearance, literature, analysis

## Abstract

Use of the terms *cognition* and *behavior* and their variants can be traced back to the middle-ages. What is not widely known is how the terms were first used in the literature. This article identifies variations of terms for cognition and behavior and traces the first use of the terms using the *Oxford English Dictionary (OED)*. A systematic search of the *OED* was conducted, identifying terms in the *cognition* and *behavior* families. Terms are defined and the year the term first appeared in the literature is identified. Terms are sorted and grouped chronologically by first appearance to determine their first use in the literature as noted in the *OED*. Results indicated more words are related to cognition than behavior. The first term related to cognition to appear was *cogitation* in *circa* 1225; while the first term related to behavior was *port*, which appeared *circa* 1330. Each family of terms experienced tremendous growth during the first appearance of terms. The cognition family saw 60% of its terms appear in the 17^th^ and 19^th^ centuries. The behavior family saw nearly 75% of its terms make their first appearance during the 15^th^ through the 17^th^ centuries.

## 1. Introduction

Psychology as a disciplinary study has seen many approaches. Two of the most dominant approaches are cognitivism and behaviorism. 

Behaviorism, championed by Watson and Skinner, focuses on observable physical and social environment and concentrates on observable and measurable behavioral-environment relationships [1]. Behaviorism advocates psychology should concern itself with observable behavior and not unobservable events occurring in the mind. 

Cognitivism, or cognitive psychology, championed by Neisser, emerged as a reaction to behaviorism. Cognitive psychology studies human mental activities, particularly information acquisition, storage, and retrieval. Cognitive psychologists study such areas as thinking, memory, perception, problem solving, intelligence, reasoning, language, and creativity [2]. 

Much debate and research has occurred concerning how the term cognitive became associated with experimental psychology [3]. Green argued “cognition was a growing concern in philosophy, artificial intelligence, and linguistics long before it caught on in experimental psychology” [3]. Cognitivism influenced nearly all areas of psychology today, and merged with many other disciplines to form the larger discipline (or “interdiscipline”) of cognitive science, which includes elements of artificial intelligence, neuroscience, philosophy and others [3]. 

What of the words, themselves; when did the terms cognition and behavior first appear in the literature? Today, as cognitivist and behaviorist approaches dominate the study of psychology, there is a significant “sibling rivalry” between the two schools of thought. The importance of the words “cognitive” and “behavior” as both strong signifiers and polarizing factors suggests an exploration of the first use of these key terms might yield interesting results and provide a starting point for a better understanding of their possible evolution. It is possible to identify variant terms that share a common stem word with cognition and behavior and look at the date those terms first appeared in the literature to see what might be revealed about those word families. This research is not concerned with tracing the changes in meaning of terms over time. It is a conversation starter and a product of intellectual curiosity regarding the first appearances of words and determining if there are patterns in those first appearances. Others may wish to pursue how the meanings of these terms changed over time.

This research might be of interest to undergraduate psychology students, researchers of the history of psychology, and others interested in the first appearances of terms, such as etymologists, geographers, *etc.*

Finally, it should be mentioned that the author is an academic librarian, serving as psychology subject specialist with more than 20 years of experience in social sciences librarianship, and comes at this research from a different perspective than would a psychologist.

## 2. Method

### 2.1. Materials

The *Oxford English Dictionary (OED)* traces a word’s history from written literature and is an accepted authority on the English language [4]. The *OED* has existed for more than 150 years, with work beginning on what would become the *OED* in 1857. The *OED*’s value is that it lists obsolete words, or words no longer used. Further, it provides dates words first appeared in written form and how they were originally used in the literature. The *OED* is an excellent resource for identifying words like cognition, behavior, and similar terms in written history.

While the *OED* tracks the earliest appearances of words, it does not and cannot tell the oral history of those words; that is, when those words were first coined or spoken. However, it does provide a starting point to investigate the history of the word’s early use. 

There are different ways to search the *OED*. Oklahoma State University’s Library reference collection contains the second edition of the *OED*—which was published in 1989—and provides access to the *OED Online*. 

As the *OED* is a British resource, all spellings and definitions for the terms are British.

### 2.2. Inclusion Criteria

To compare the cognition and behavior families of terms throughout history, a starting point for an analysis must be determined. It makes sense to look to the earliest root words for cognition and behavior and use those root words as a springboard to identify and list members of the family of terms.

The *Oxford English Dictionary (OED)* notes cognition traces its roots to the Latin *cognit-*, (“a getting to know, acquaintance, notion, knowledge, *etc.*”). Eric Partridge’s *Origins: A Short Etymological Dictionary of Modern English* notes cognition originates from the Latin *cognōscere* (“to know, to learn about”). Cognōscere is a compound of *co-* (“together”) and *gnōscere*, an early form of *nōscere* (“to know”) [5]. 

More etymology comes from the *Etymological Dictionary of Latin and the other Italic Languages*, which notes the verb *cogito/cogitare* (“I know/to know”) is made from the prefix *co-* (“with” or “together”). Add to that the verb *agitare* (“to stir, drive, disturb, be occupied, aspire to”), which comes from *ago/agree *(“to drive” or “to move”) [6]. Basically, it means to shake things or put things together. 

The root word for behavior is *behave*. The *OED* etymology notes that behave formed in the 15^th^ century from the prefix *be-*; plus *have*, which means “in order to express a qualified sense of have, particularly in the reflexive ‘to have or bear oneself (in a specified way)’.” 

As terms related to cognition trace back to the Latin *cognōscere/cogitare*, so does the word behave. The Latin counterpart to behave is *com+portare*, which can loosely translate to mean how you carry yourself. *Com-*, which means “ready, completely; with”, and *portare*, which means “to transport, carry” [7].

When considering terms based on cognition, behavior, and their variants, terms such as *incognito, recognize,* and *misbehavior* come to mind. Any search to identify terms in the cognition and behavior families should include variations that start with different Greek and Latin prefixes. The common Greek and Latin prefixes to be used to identify term variants includes the following: *a-*, *an-*, *anti-, de*-, *dis*-, *em*-, *en*-, *fore*-, *i*-, *im*-, *in*-, *inter-*, *intra-*, *ir-, mid-, mis-, non-, over-, pre-, re-, semi-, sub-, super-, trans-, un-, *and *under-*.

To be included in the cognition or behavior families of terms a systematic search of the *OED* will be employed to identify terms which contain cognition and behavior root words: *cogit* or *cognit* (cognition) and *behave* or *comport* (behavior). Further, term variants of the root words were identified by expanding the *OED* search to include common Greek and Latin prefixes that might precede either *cogni, cogit, behave* and *comport*. 

### 2.3. Procedure

A systematic search of the *OED* was employed to identify *cognition *(*cognōscere/cogito/cogitare*), *behavior* (*behave/comportare*), and related terms (words spelled similarly or that sound the same). To identify term variants that start with different prefixes, a list of common Greek and Latin prefixes was searched in the *OED*. 

To identify variants of the root words in the *OED*, a search strategy was employed to pair the prefix with the root word. For example, the *OED* was searched for *acognit*, *ancognit*, *anticognit*, and so on, working through the list of common Greek and Latin prefixes to identify variants. 

After generating a list of terms in the “cognition family” and the “behavior family,” the *OED Online* was searched to provide definitions for each term and the year the term first appeared in the literature. 

Definitions for the terms are provided to understand the context for the term’s first use. The OED lists terms that could be obsolete, old, or no longer being used, or used in the way they are today. Definitions are listed purely for context. 

The year the word was first used in the literature is relevant to term-use history investigation. The behavior and cognition family word lists were entered into a Microsoft Excel spreadsheet to facilitate organization and sorting. The terms identified for the cognition and behavior families were sorted by the year the term first appeared and categorized by century to track when the terms appeared. Calculations were performed on the groups to determine percent of terms appearing by century.

## 3. Results and Discussion

The research on the word cognition began by consulting the third volume of the *OED*, which contains cognition. After listing each term containing the stem words *cognōscere and cogito/cogitare*, further searching was conducted for variations with different Greek and Latin prefixes, resulting in an alphabetical listing. This listing is the cognition family of terms and it appears below. 

**Table:** 

Cogitability	Incogitable	Precognizance
Cogitable	Incogitability	Precognizant
Cogitabund	Incogitance	Recogitate
Cogitancy	Incogitancy	Recogitation
Cogitandum	Incogitant	Recognition
Cogitant	Incogitantly	Recognitive
Cogitate	Incogitate	Recognitor
Cogitation	Incogitative	Recognitory
Cogitative	Incognitive	Recognizability
Cogitativity	Incognita	Recognizable
Cogitatum	Incognite	Recognizably
Cogito	Incognito	Recognizance
Cognition	Incognizability	Recognize
Cognitional	Incognizable	Recognized
Cognitive	Incognizance	Regognizee
Cognitively	Incognizant	Recognizer
Cognitivist	Incognoscible	Recognizon
Cognitor	Incognoscibility	Recognizor
Cognitum	Miscognizant	Recognosce
Cognizable (-sable)	Miscognize	Uncogitable
Cognizably	Praecognitum	Uncognisant
Cognizance (-sance)	Precog	Uncognizable
Cognizant (-isant)	Precogitancy	Uncognized
Cognize (-ise)	Precogitate	Uncognoscibility
Cognizee (-isee)	Precogitation	Uncognoscible
Decognize	Precognit	
Discognisance	Precognition	

There are 79 terms identified as the cognition family. After the list of cognition family terms was compiled, definitions from the *OED* and the date the term first appeared in the literature were added. The list of cognition family terms was sorted chronologically by the year the term first appeared to create a new list. This appears in Table 1. 

**Table 1:** Cognition words defined and listed by date.

Date of First Appearance	Term	Definition
*Circa* 1225	Cogitation	The action of thinking or reflecting; attentive consideration, reflection, meditation
1325	Recognizance	A bond or obligation by which a person undertakes before a court or magistrate to perform some act or observe some condition
1388–89	Recognize	Of a feudal superior: to resume possession of land
1394	Cognizant (-isant)	To know, recognize
1400	Cognizance (-sance)	Knowledge, understanding; also acquaintance
1400–1	Recognosce	Of a feudal superior; to resume possession of (land)
1436	Recognizor	One who enters into a recognizance
1447	Cognition	The action or faculty of knowing; knowledge, consciousness; acquaintance with a subject
1450	Recognition	Knowledge or consciousness; understanding
1460	Recognitor	A member of a jury impanelled on an assize or inquest
1477	Discognisance	Non-recognition
1490	Cogitative	Having the power or faculty of thought; thinking (as a permanent attribute)
About 1500	Precognition	Antecedent cognition or knowledge; (supposed) foreknowledge, esp. as a form of extrasensory perception
1522	Incogitable	Unthinkable, inconceivable
1529	Uncogitable	Obsolete word
1531–32	Cognizee (-isee)	The party in whose favour a fine of land was levied; he to whom cognizance was made
1540	Miscognizant	Not cognizant, knowledgeable, or aware; ignorant of something; spec. ignorant of the law, or some aspect of it
1569	Precogitate	To cogitate, think, or think over beforehand; to consider beforehand, premeditate
1586	Cognitive	Of or pertaining to cognition, or the action or process of knowing; having the attribute of cognizing
1591	Recogitation	The action or result of thinking over something again; an instance of this
1592	Recognizee	The person to whom another is bound in a recognizance
1596	Precogitation	Previous consideration or meditation; thinking over beforehand; a prior reflection or idea
1602	Recogitate	To think over (something) again
1603	Miscognize	To fail to appreciate or acknowledge
1608	Recognizer	A person who (or occasionally a thing which) recognizes someone or something
1609	Incognite	Unknown
1611	Recognizon	Acknowledgement
1612	Incogitancy	Want of thought or reflection; thoughtlessness, heedlessness, negligence; inadvertence
1624	Praecognitum	A thing already known, especially a thing needed or assumed to be known in order to infer or ascertain something else
1628	Incogitant	Thoughtless, unthinking; characterized by want of thought; inconsiderate
1633	Cogitate	To think, reflect, ponder, meditate; to exercise the thinking faculties
1637	Incogitance	Want of thought
1638	Incognito	An unknown man; one who conceals his identity; an anonymous person
About 1645	Precogitancy	Prior consideration or thought
1648	Incogitantly	Unthinkingly, thoughtlessly, without consideration or reflection
1649	Cogitabund	Musing, meditating, thoughtful, deep in thought
1652	Incogitate	Not thought of; unexpected; unpremeditated
1654	Precognit	A preliminary discussion
1659	Cognize (-ise)	To take cognizance
1659	Decognize	To cease or fail to recognize
1671	Incognita	A feminine version of incognito
1678	Cognizable (-sable)	Capable of being known, perceived, or apprehended by the senses or intellect; perceptible
1680	Cogitant	Thinking, that thinks
1682	Recognizable	Able to be recognized or identified; that permits recognition
1688	Cogitability	Capability of being thought or conceived
1688	Cogitable	That can be thought or conceived; thinkable, conceivable
1690	Incogitative	Unthinking; destitute of the thinking faculty
1691	Incognoscible	Unknowable, beyond cognizance
1720	Uncognizable	
1722	Cogitativity	Capacity or power of thinking
1726	Precognizance	Prior knowledge or understanding
1759	Cogitancy	Cogitant or thinking quality
1790	Recognized	Acknowledged, accepted; known, identified
1802	Recognitive	Of, relating to, or involving recognition or acknowledgment; that recognizes
1813	Recognitory	Of, or relating to, recognition or acknowledgement
1817	Cognizably	In a cognizable manner; recognizably; perceptibly
1821	Uncognoscible	
1824	Incognoscibility	The quality or condition of being incognoscible; unknowableness
1827	Cognitional	Of or pertaining to cognition
1827	Uncognoscibility	
1831	Recognizably	To a recognizable degree, perceptibly; in a recognizable manner
1836	Recognizability	The fact or quality of being recognizable
1837	Incognizant	Not cognizant; without cognizance, knowledge, or apprehension of; unaware, unconscious of
1840	Precognizant	Having previous cognizance; having prior knowledge or understanding (of something)
1852	Incognizable	Not cognizable; incapable of being known, perceived, or apprehended by the senses or intellect; incapable of recognition
1853	Incogitability	The quality of being unthinkable; incapability of being thought
1854	Cogito	The principle ‘cogito, ergo sum’, or any equivalent formula, by which Descartes claimed to establish his own existence as a thinking being from the fact of his thinking or awareness; loosely, conscious awareness or subjectivity
1856	Incognizance	Want of knowledge or recognition
1860	Incognizability	The quality of being incognizable
1860	Uncognisant	
1862	Incognitive	Destitute of the faculty for cognition; unable to take cognizance
1866	Cogitandum	That which should be thought; the ideal or correct processes of thought, as opposed to the actual processes
1875	Cognitum	An object of cognition
1877	Uncognized	
1878	Cogitatum	That which is thought; the actual processes of thought, as opposed to the ideal thought-processes
1880	Cognitively	In a cognitive manner; with regard to, or from the point of view of, cognition
1880	Cognitor	An attorney or procurator
1952	Cognitivist	One who holds that moral judgments are true or false statements about moral facts
1954	Precog	A person who predicts something; a person with precognition

The earliest appearance of a term from the cognition family is *circa* 1225 with the word *cogitation*. The next two terms on the list, *recognizance* and *recognize* have more to do with feudal society than thinking. The *OED* defines terms as they were originally used in the literature.

Having identified, defined, and sorted a list of terms in the cognition family, attention was turned to the behavior family. For purposes of accurate comparison to cognition, this search looked at terms like *behave* and *comport*. The terms identified as a part of the behavior family are listed below.

**Table:** 

Behave	Comportance	Deportee
Behaved	Comportation	Deportment
Behaving	Comportioner	Deporture
Behaviour	Comportment	Incomportable
Behavioural	Deport	Interbehaviour
Behavioured	Deportable	Misbehave
Behaviourism	Deportate	Misbehaved
Comport	Deportation	Misbehaviour
Comportable	Deportator	Port

The behavior family contains 27 terms, far fewer than terms in the cognition family. Again, the *OED* was consulted for definitions and the year the terms first appeared in the literature. This listing appears in Table 2. 

The first term in the behavior family to appear in the literature chronologically was *port*, which appeared *circa* 1330, not long after the first term in the cognition family first appeared.

Both chronological lists of terms were sorted by date and grouped into categories by century. Calculations were performed to determine by century the percent of terms appearing for the first time. This is featured in Table 3.

**Table 2:** Behavior words, defined and listed chronologically.

Date of First Appearance	Word	Definition
*Circa* 1330	Port	Bearing, deportment, or carriage, esp. dignified or stately bearing; demeanour or manner
*Circa* 1440	Behave	To bear, comport, or conduct oneself; to act
1474	Deport	Behavior, bearing, deportment
1475	Misbehave	To behave badly or wrongly; to conduct oneself improperly
1482	Behaving	Conduct, behaviour
1486	Misbehaviour	Bad behavior, improper conduct
1490	Behaviour	Manner of conducting oneself in the external relations of life; demeanor, deportment, bearing, manners
1588	Comport	To bear, endure; to tolerate
1589	Behavioured	Conducted, mannered, behaved
1590	Comportance	Carriage, bearing, behaviour, manner of conducting oneself
1595	Deportation	The action of carrying over; forcible removable, especially into exile
1597	Misbehaved	Badly behaved
1599	Comportable	Capable of being borne or endured; tolerable, bearable
1599	Deportate	To carry or convey away
1601	Deportment	Manner of conducting oneself; conduct (of life); behavior
1604	Behaved	Conducted, mannered
1605	Comportment	Personal bearing, carriage, demeanor, deportment; behaviour, outward conduct, course of action
1609	Comportioner	One of a number who share together
1611	Deporture	Carriage, bearing, deportment
1616	Deportator	One who deports or transports
1633	Comportation	The action of bringing together or collecting
About 1734	Incomportable	Not to be borne, intolerable, insupportable
1891	Deportable	Liable to, or punishable by, deportation
1895	Deportee	One who is or has been deported
1913	Behaviourism	A theory and method of psychological investigation based on the study and analysis of behaviour
About 1927	Behavioural	Concerned with, or forming part of, behaviour
1939	Interbehaviour	

**Table 3:** Cognition and Behavior terms categorized by century of first literary appearance.

Century	Cognition Words that Make Their First Appearance		Behavior Words that Make Their First Appearance	
	n	%	n	%
13^th^	1	1.26	0	0
14^th^	3	3.79	1	3.70
15^th^	8	10.12	6	22.22
16^th^	10	12.65	7	25.92
17^th^	26	32.91	7	25.92
18^th^	5	6.32	1	3.70
19^th^	24	30.37	2	7.40
20^th^	2	2.53	3	11.11

The research results found in Table 3 are interesting on a couple of levels. 

First, it revealed some centuries are characterized by tremendous numbers in terms of the initial appearances of terms, starting in the 15^th^ century. Seventy-nine terms are part of the cognition family, *versus* 27 terms in the behavior family. In terms of a breakdown within each family of terms, the Latin stem word *cognōscere* spawned 53 terms, while the stem word *cogito/cogitare* spawned 26 terms. In the behavior family, the stem word *behave* spawned 11 terms, while the stem word *comportare* spawned 16 terms. Why there are so many words in the cognition family as opposed to the behavior family is an area for other researchers to investigate.

Second, the cognition family saw 62.91% of the terms make their initial appearance in the literature in just two centuries—the 17^th^ and the 19^th^ centuries. On the other hand, during three consecutive centuries, the 15^th^ through the 17^th^ centuries, the behavior family saw nearly 75% of its terms appear in the literature.

Why do these centuries account for such a large percentage of these term’s first appearances? An initial explanation is that there were more texts available for inclusion in the *OED*. The *OED* can only include existing texts available for analysis. Johannes Gutenberg invented the first moveable type printing press in the 1440s, during the fifteenth century [8]. Prior to Gutenberg’s printing press, books were copied by hand, a far more laborious and expensive process, which made texts less likely to survive and consequently harder to find. Gutenberg’s invention enabled mass, quick, and cheap book production, which meant more books available for analysis in the *OED*. Thus it is no surprise that more words appear for the first time in the literature starting in the 15^th^ century.

The 17th century was the advent of the Age of Enlightenment or simply the Enlightenment, also the Age of Reason. The Enlightenment started in Europe and eventually spread to the United States. It started generally in the last decade of the seventeenth century and lasted as late as the French Revolution, *circa* 1800. The Enlightenment was an intellectual movement which sparked a curiosity about mankind and the world and more attention to learning and knowing [9]. During the 19^th^ century, psychology became a unique scientific discipline separate from its philosophical roots. John G. Benjafield, in his book *Psychology: A Concise History* traces the history and development of psychology and notes that in the nineteenth century, through the work of influential scholars Fechner, Galton, and others, psychology developed into a truly scientific discipline [10]. It is possible terms for cognition occurred during this century to support establishing psychology as a discipline. It is into this environment the cognition and behavior families of terms perhaps appeared.

## 4. Conclusions

From the data compiled, more terms pertain to cognition than to behavior. Cognition terms trace their first appearance back to as early as *circa* 1225, when *cogitation* appeared for the first time in the literature. Meanwhile, the behavior family saw its first term, *port*, appear about a century after *cogitation*. Further, each family of terms experienced tremendous growth in the initial appearances of terms. The cognition family saw more than 60% of its words appear in two centuries—the 17^th^ and the 19^th^ centuries. The behavior family saw nearly 75% of its words appear in the literature for the first time in the 15^th^ through the 17^th^ centuries. 

Because this research is intended as an opening for a dialog, it does not include answers to why these terms appeared when they did, or why the terms evolved over time, although that is something other researchers may wish to investigate. The next phase in this investigation might be to trace how the meanings of these terms have changed over time in the field of psychology.
